# Identification of marker genes in Alzheimer's disease using a machine-learning model

**DOI:** 10.6026/97320630017348

**Published:** 2021-02-28

**Authors:** Inamul Hasan Madar, Ghazala Sultan, Iftikhar Aslam Tayubi, Atif Noorul Hasan, Bandana Pahi, Anjali Rai, Pravitha Kasu Sivanandan, Tamizhini Loganathan, Mahamuda Begum, Sneha Rai

**Affiliations:** 1 Department of Biotechnology, School of Biotechnology and Genetic Engineering, Bharathidasan University, Tiruchirappalli - 620024, Tamil Nadu, India; 2 Department of Computer Science, Faculty of Science, Aligarh Muslim University, Aligarh - 202002, Uttar Pradesh, India; 3 Faculty of Computing and Information Technology, Rabigh, King Abdulaziz University, Jeddah - 21589, Kingdom of Saudi Arabia; 4 Department of Computer Science, Jamia Millia Islamia (Central University), Jamia Nagar - 110025, New Delhi, India; 5 Department of Bioinformatics, Sambalpur University, Jyoti Vihar, Burla, Sambalpur - 768019, Odisha, India; 6 Department of Biotechnology and bioinformatics, Mahila Maha Vidyalaya , Banaras Hindu University, Varanasi - 221005, Uttar Pradesh, India; 7 Department of Bioinformatics, School of Biosciences, Sri Krishna Arts and Science College, Coimbatore - 641008, Tamil Nadu, India; 8 Department of Biotechnology, Bhupat and Jyoti Mehta School of Biosciences, IIT Madras and Initiative for Biological Systems Engineering (IBSE), Chennai - 600036, Tamil Nadu, India; 9 PG and Research Department of Biotechnology, Marudhar Kesari Jain College for Women, Vaniyambadi - 635751, Tamil Nadu, India;; 10 Department of Biological Sciences and Engineering, Netaji Subhas Institute of Technology, Dwarka - 110078, New Delhi, India

**Keywords:** Alzheimer's Disease, Biomarkers, In-silico Analysis, Machine Learning, Cross-validation, Classifiers, Bayes Net, Naive Bayes, Decision Table, J48, SMO/SVM, Log it Boost

## Abstract

Alzheimer's Disease (AD) is one of the most common causes of dementia, mostly affecting the elderly population. Currently, there is no proper diagnostic tool or method available for the detection of AD. The present study used two distinct data sets of AD genes, which could be potential biomarkers in the diagnosis. The differentially expressed genes (DEGs) curated from both datasets were used for machine learning classification, tissue expression annotation and co-expression analysis. Further, CNPY3, GPR84, HIST1H2AB, HIST1H2AE, IFNAR1, LMO3, MYO18A, N4BP2L1, PML, SLC4A4, ST8SIA4, TLE1 and N4BP2L1 were identified as highly significant DEGs and exhibited co-expression with other query genes. Moreover, a tissue expression study found that these genes are also expressed in the brain tissue. In addition to the earlier studies for marker gene identification, we have considered a different set of machine learning classifiers to improve the accuracy rate from the analysis. Amongst all the six classification algorithms, J48 emerged as the best classifier, which could be used for differentiating healthy and diseased samples. SMO/SVM and Logit Boost further followed J48 to achieve the classification accuracy.

## Background

Alzheimer's Disease (AD), a cognitive, neurological disorder characterized by progressive dementia, commonly causes dementia. Pathologically, AD is marked by degeneration of myelinated axons of nerve cells, the presence of neuritic plaques, and neurofibrillary tangles (NFT) [1]. AD evolves epidemically within the population in their mid to advance age currently, no specific therapy and technique are available for treatment and detection of AD, respectively. The presence of progressive dementia is considered as one of the prominent diagnostic features of AD when there is no sign of other neurological disorders such as Parkinson's disease, drug intoxication, manic-depressive illness and pernicious anemia, chronic infections of the nervous system, Huntington's disease and brain tumor [2]. The other plausible ways of diagnosing AD is by examining a patient's medical and clinical history [2]. The most common clinical tests used to detect AD are NMR/MRI, electrophysiologic method, positron emission tomography (PET) and regional cerebral blood flow [3]. However, the unprecedented growth of scientific ability and knowledge has left behind the lagging retro diagnosis techniques. Modern Techniques of AD diagnosis include the usage of fluid biomarkers detected by structural MRI and cerebrospinal fluid analysis and neuroimaging techniques such as molecular neuroimaging with PET. These modern techniques are capable of detecting early and significant memory dementia [4,5]. However, a definite confirmation of AD is still dependent on pathological analysis at autopsy [6]. To date, researchers have made a significant contribution in developing biomarkers for the detection of AD. These biomarkers provide an easy, less invasive and more accurate diagnosis of AD [7]. Apolipoprotein (APOE) is one of the most prominent biomarkers of AD and its polymorphism is associated with the risk of AD progression [8,9]. TOMM40 gene with amyloid-beta negatively impacts the downstream apoptotic process. Therefore TOMM40 is related to the new-onset of AD [10,11]. The amyloid-beta formation is associated with the alteration of the amyloid precursor protein, leading to the deregulation of the gene APP that results in the early-onset of AD [12]. There are two critical genes, Presenilin 1(PSEN1) and Presenilin 2 (PSEN2) that help regulate the amyloid cascade. These genes are also considered as susceptible genes, resulting in the late onset of AD [13,14]. Moreover, low expression of SORL1 promotes the overexpression of beta-amyloid, thereby the risk of AD increases [15]. Neurodegeneration results from aberrant cell cycle activity in neurons [16], which progressively affects the limbic and cortical brain regions. The cell cycle's abnormal movement disrupts the various cognitions related to memory, emotional learning and perception. Transcriptional analysis of cell cycle regulation in several organisms has originated from the relation of genes in regulating the cell cycle [17,18, 19]. Microarray-based studies have been considerably identified as remarkable biomarkers not limited to AD but in other disease complexities [20,21, 22]. The present research objective was to identify the most suitable set of genes that helps in the progression of Alzheimer's Disease, utilizing Machine learning classifiers such as Bayes Net, Naïve Bayes, SMO/SVM, Logit Boost, Decision Table and J48. The percentage accuracy was measured by using twenty-fold cross-validation for each classifier. The SMO/SVM, Log it Boost and J48 were identified as the most accurate classifiers, which resulted in 90% of accuracy. Recent studies have also supported the accuracy of SVM and J48 algorithms for AD sample classification [23,24].

## Methodology

### Data and data Source:

Two different Gene expression datasets analyzed on the HG-U133_Plus_2 platform were retrieved from NCBI's GEO database (https://www.ncbi.nlm.nih.gov/geo/). The first dataset (Accession ID: GDS2795) was collected from samples of Neurofibrillary tangles bearing entorhinal cortex (Diseased) and Non-neurofibrillary tangles bearing entorhinal cortex (Non-diseased/Normal). The second dataset (Accession ID: GDS4136) had samples from Hippocampal sections (CA1) tissue blocks containing gray and white matters. These samples were classified as Control, Incipient, Moderate and Severe. Only Control/Normal and Severe samples were selected for further analysis.

### Data processing and DEGs extraction:

The normal and diseased samples from both the datasets were downloaded in CEL format and analyzed in R (4.0.3) utilizing Bioconductor packages. The probe intensities were normalized using RMA package and DEGs were obtained using limma package of Bioconductor. The p-value for the two datasets was set to 0.01 and 0.001, respectively, to obtain DEGs top-hits.

### Machine learning classifier and DEG Cross-Validation:

**Figure 1:**
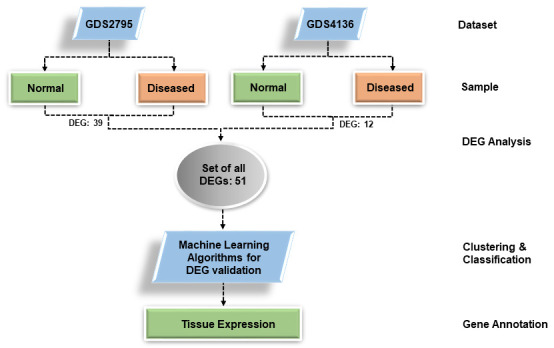
Overview of experimental design: The experiment begins with data sets selection followed by DEGs extraction and their validation through machine learning classifiers. Their tissue expression annotation further validated DEGs.

Machine learning classifier and cross-validation of DEGs were performed in Weka (Waikato Environment for Knowledge Analysis). Weka is open-source software that helps in data preprocessing and implementing several Machine Learning algorithms to solve real-world data complexities by clustering, classification and other techniques. The DEGs obtained from both the datasets were taken and their transformed expression values respective to each sample type were fed to six different classifiers. For classification, samples were categorized into two classes i.e. Normal and Diseased. Twenty folds cross-validation were set with each classifier. The classifiers used were Bayes Net, Naive Bayes, SMO/SVM, Logit Boost, Decision Table and J48. Figure 1 shows the workflow of the analysis.

### Tissue expression annotation:

An online functional annotation tool DAVID was used for identifying the expression of DEGs in their respective tissues. Further, DEGs were fed to Gene Mania® online tool to identify their co-expression and evaluate its association with other genes with the help of co-expression GRN.

## Results:

### DEGs extraction and gene annotation:

A total of 39 genes and 12 genes were obtained from GDS2795 and GDS4136, respectively, data presented in Table 1. Total 38 genes were found to be annotated in DAVID. Genes such ASGPR84, LMO3, N4BP2L1, ST8SIA4, PSITPTE22, CNPY3, CDK6, DTNA, GNAL, HIST1H2AB, HIST1H2AE, LOC339047, ZNF337, LOC100132540, IFNAR1, LRRC8A, METTL14, MYO18A, NLGN1, LOC652346, PML, SEMA6A, TLE1, LOC645382, SLC4A4 and ZNF518A were expressed in the brain, data shown in Table 2.

### Machine learning:

Among all the classifiers, only six classifiers showed the highest accuracy with 90%, later followed by 85% accuracy. The True Positive Rate (TP Rate) and False Positive Rate (FP Rate) for these classifiers varied from 0.9 to 0.8 and 0.0 to 0.2, respectively. The accuracy percentage of SMO/SVM, Log it Boost and J48 was 90%, whereas the accuracy percentage of Naïve Bayes, Bayes net and Decision Table was 85%. Table 3 representing the classification results from all these classifiers.

### Co-expression GRN and Co-expressed genes:

**Figure 2:**
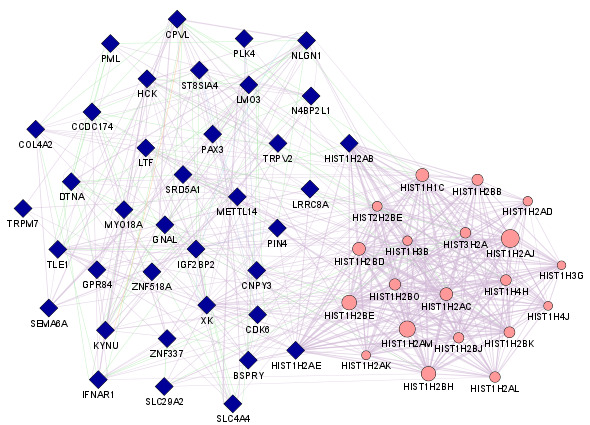
The GRN co-expression: co-expression association of query genes (DEGs) with other genes. The blue pointers are query genes, and pink pointers genes co-expressed with the query gene suggested by the tool.

The co-expression GRN of the DEGs was obtained from both the data sets shown in Figure 2. From all the DEGs, the Gene Mania tool did not recognize 14 DEGs and the remaining 36 DEGs were used as query genes for co-expression GRN construction. Twenty query genes were found to be in co-expression association with other genes, including query and non-query genes. Table 3 representing co-expressed query genes with their respective co-expressed genes.

## Discussion:

**Figure 3:**
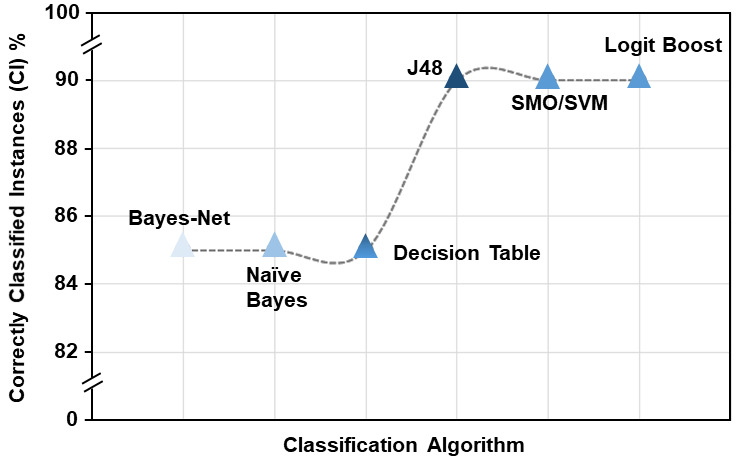
Performance of accuracy for each classifier of the 20 rounds.

Two different datasets of gene involved in AD progression were used in identifying DEGs. Different p-values, e.g., 0.01 and 0.001, were used to generate top genes with higher differential expression. We have identified a total of 51 DEGs, 39 and 12 from GDS2795 and GDS4136, respectively. Further, validation was performed for assessing the involvement of DEGs in AD. These genes were subjected to the online annotation tool DAVID. The genes obtained from the annotation tool were GPR84, LMO3, N4BP2L1, ST8SIA4, PSITPTE22, CNPY3, CDK6, DTNA, GNAL, HIST1H2AB, HIST1H2AE, LOC339047, ZNF337, LOC100132540, IFNAR1, LRRC8A, METTL14, MYO18A, NLGN1, LOC652346, PML, SEMA6A, TLE1, LOC645382, SLC4A4 and ZNF518A, and these 26 genes are expressed in the brain. Further, the next hypothesis was to identify whether these genes could be implemented to classify a sample as Diseased or Normal. For attaining this objective, the training data set was prepared by using 26 genes and twenty-fold cross-validation was utilized. As a result, classifiers such as SMO/SVM, J48 and Log it Boost achieved 90% accuracy, while Naïve Bayes, Bayes Net and Decision Table attained only 85% accuracy. Since machine-learning classifiers have been widely used for sample classification [25,26, 27], our classifiers' accuracy confirms the studies where the identified final DEGs could aid in differentiating Normal and AD samples. The co-expression analysis data revealed that 20 genes out of 51 DEGs were co-expressed with other genes. According to our results, these DEGs are associated in the co-expression with the other genes and also expressed in the brain tissue: CNPY3, GPR84, HIST1H2AB, HIST1H2AE, IFNAR1, LMO3, MYO18A, N4BP2L1, PML, SLC4A4, ST8SIA4, TLE1 and N4BP2L1. It is considered that TPR nearly 1.00 and FPR close to 0.00 are best for any classification result, which uses any classifier [28,29]. In our study, the highest and lowest TPR were 0.9 and 0.8, respectively. Bayes Net, SMO/SVM, Log it Boost and Decision Table have the highest TPR, whereas the lowest FPR was 0.0, achieved by the J48 classifier. Among all the classifiers, J48 could be concluded best to provide outcome with 90% accuracy of CCI %, 0.1 True Positives (TP) and 0.0 False Positive (FP) rates. Figure 3 shows the average accuracy performance results in 20 folds cross-validation for the considered classifiers (Bayes Net, Naïve Bayes, SMO/SVM, Log it Boost, Decision Table and J48).

## Conclusion

In the present study, an integrated approach of bioinformatics data analysis and machine learning classification was used. We have identified 13 DEGs (CNPY3, GPR84, HIST1H2AB, HIST1H2AE, IFNAR1, LMO3, MYO18A, N4BP2L1, PML, SLC4A4, ST8SIA4, TLE1 and N4BP2L1) that could be utilized in distinguishing AD and Normal samples. Therefore, these 13 genes could be used as potential gene set as biomarkers to identify AD. Moreover, only six machine-learning classifiers qualified for further analysis and J48 emerged as the best classifier amongst all the classifiers. The accuracy of J48 was 90% and TPR was found to be 0.9 and 0.00, respectively. Other classifiers such as SMO/SVM and Log it boost showed an accuracy of 90% and attained TPR and FPR 0.9 and 0.0, respectively. Therefore, the results from this study also signify the highest accuracy result from J48 algorithm amongst the set of six considered classifiers applied on the same data. This accuracy of J48 for sample classification may need further validation by using it to datasets from a broader range of AD samples and other diseases.

## Figures and Tables

**Table 1:** Differentially Expressed Genes in GDS2795 and GDS4136

GDS 2795	GDS4136
MYO18A	METTL14	ST8SIA4	CPVL	KYNU	LMO3	LOC157562	LTF
LOC286154	COX2	SLC4A4	SEMA6A	IGF2BP2	TRPM7	PIN4	NLGN1
GPR84	CNPY3	N4BP2L1	IFNAR1	CDK6	BSPRY	HCK	LOC728485
MGC24125	LOC645381	LOC255025	SLC29A2	COL4A2	LRRC8A	LOC643201	ZNF337
HIST1H2AB	TLE1	LOC339047	TRPV2	PAX3	PML	CCDC174	SRD5A1
HIST1H2AE	PSITPTE22	LOC100132540	LOC652346	PLK4	GNAL	ZNF518A	NA (215816_AT)
NA: Not Available (gene symbol)

**Table 2:** 26 genes were found to be expressed in brain tissues and other tissues from DEG tissue Expression data

GENE	TISSUE EXPRESSION	GENE	TISSUE EXPRESSION
GPR84	Brain	LOC100132540	Brain, Cerebellum, Umbilical cord blood
LMO3	Brain	IFNAR1	Brain, Liver, Myeloma, Ovary
N4BP2L1	Brain	LRRC8A	Brain, Epithelium, Pancreas
ST8SIA4	Brain, foetal brain, Lung	METTL14	Brain, Lung, Muscle
PSITPTE22	Hippocampus,	Myo18a	Brain, Epithelium, Liver, Testis
CNPY3	Brain cortex, Cervix, Colon, Liver	NLGN1	Brain, Duodenum, Embryo
CDK6	Brain, Tongue	LOC652346	Brain, Epithelium, Kidney, Spleen
DTNA	Brain, foetal brain, Heart	PML	Brain, Epithelium, Kidney, Spleen
GNAL	Amygdala, Brain, Hippocampus, Insulinoma, Testis	SEMA6A	Brain, Hypothalamus, Placenta
HIST1H2AB	Brain, Liver	TLE1	Aorta endothelial cell, Colon, Foetal brain, Kidney
HIST1H2AE	Brain, Liver	LOC645382	Aorta endothelial cell, Colon, Foetal brain, Kidney
LOC339047	Brain, Cerebellum, Umbilical cord blood	SLC4A4	Brain, Heart, Pancreas, Prostate, kidney
ZNF337	Brain, Lung,	ZNF518A	Brain, Epithelium, Lung, Retina,

**Table 3:** Classification results for six classifiers and their accuracy for correctly classifying the sample types

Classifiers	CCI (%)	ICI (%)	TP rate	FP rate
Bayes Net	85	15	0.9	0.2
Naïve Bayes	85	15	0.8	0.1
Decision Table	85	15	0.9	0.2
J48	90	10	0.9	0
SMO/SVM	90	10	0.9	0.1
Logit Boost	90	10	0.9	0.1
